# Evolving market-shaping strategies to boost access to essential medical products in developing countries with HIV self-testing as a case study

**DOI:** 10.1186/s41256-023-00310-5

**Published:** 2023-07-14

**Authors:** Jian Yang, Xiangning Feng, Shuduo Zhou, Li Zhang, Yunxuan Hu, Ying Chen, Zhenyu Zhang, Ming Xu

**Affiliations:** 1 Department of Global Health, School of Public Health, Peking University, 38 Xue Yuan Road, Haidian District, Beijing, 100191 China; 2 Institute for Global Health and Development, Peking University, Beijing, China; 3 Asia Pacific Medical Technology Association (APACMed), Singapore, Singapore

**Keywords:** Market-shaping, Essential medical products, HIV self-testing, Developing countries

## Abstract

The COVID-19 pandemic has exacerbated health inequities among countries in the Global South with limited access to essential medical products, leading to a higher infection and mortality rate, especially among vulnerable populations. Despite tremendous progress in global health financing, the estimated annual financing gap in developing countries is projected to reach US$371 billion per year by 2030. Therefore, developing market-shaping strategies is of great importance in ensuring adequate supply, affordable prices, and equitable access to essential medical products in low-and middle-income countries. We propose a strategic and appropriate market-shaping intervention framework for governments, international organizations, and NGOs to maximize access to essential medical products in developing countries. In the health field, we believe that market shaping strategy could be defined as a set of purposeful activities that market forces may intervene with to advance the development, production, supply, and distribution of global goods for health, making essential medical products more affordable, accessible, innovative, sustainable and quality assured. We argue that when designing a market-shaping strategy, policy or decision-makers must take full advantage of the key drivers to keep the market dynamic, interactive, and constantly evolving to meet the unmet medical needs. In addition, different forms of market-shaping interventions are determined by objectives and specific issues to be addressed. More comprehensive market shaping strategies, including the strategic use of market expansion, market disruption, market maintenance, and market contraction alone or together, deserve to be explored and key stakeholders are also expected to join forces to make the intervention more efficient and productive.

## Introduction

The COVID-19 pandemic has exacerbated health inequities among countries in the Global South with limited access to essential medical products, leading to a higher infection and mortality rate, especially among vulnerable populations [1, 2]. It makes the dilemma of underinvestment in essential medical products for developing countries more serious. Normally, developing countries have the following channels to access essential medical products: through grants provided by international organizations, multilateral partnerships, and philanthropic foundations, such as United Nations Children's Fund (UNICEF), United Nations Population Fund (UNFPA), the Global Fund to Fight AIDS, Tuberculosis and Malaria (Global Fund), Gavi, the Vaccine Alliance (Gavi, previously the Global Alliance for Vaccines and Immunization) and the Bill and Melinda Gates Foundation; through bilateral assistance from sovereign states, and domestic health financing. In recent years, international organizations, multilateral partnerships, and philanthropic foundations have played an increasingly important role as major contributors against the backdrop that the global health community has made greater efforts to raise funds for global public goods, with the total volume of global health financing reaching $55 billion in 2020, more than four times the amount in 2003 [3]. Despite tremendous progress in global health financing, an additional $274 billion spending on health is needed per year by 2030 to make progress towards the Sustainable Development Goal 3 (progress scenario), whereas US$371 billion would be needed to reach health system targets in the ambitious scenario [4]. It is clear that the progress made to date in improving health outcomes and reducing health inequities is at risk due to underinvestment in essential medical products for developing countries, and without additional funding and effective market-shaping interventions, developing countries with high disease burden will remain vulnerable to both Current and future pandemics. Moreover, market-based systems cannot deliver essential epidemic countermeasures in a timely, fair, equitable, and sustainable manner [5]. The market forces that deliver life-saving or life-enhancing products only sometimes serve low-income countries well due to uncertain funding and demand, and unaffordable prices. And there are always market failures associated with global public goods for health. As a result, developers may not see enough patient demand to develop a new product, manufacturers may not know how much to produce, distributors may not see enough profit to justify delivery, and the purchaser is unable to procure the right product at the right price [6]. However, an appropriate market-shaping strategy, which a company usually undertakes to increase its competitiveness and create new opportunities, could be used to solve market failures [7]. The term "market shaping" has been used to refer to the phenomenon of how markets are shaped by individuals, organizations, or market forces [8]. Most recent research on market shaping has focused on describing market changes and market dynamics rather than on the market shaping itself [9], which is also defined as a cycle of interrelated actions, such as market information, market structure, and market intervention [10]. In the health field, we believe that market shaping strategy could be defined as a set of purposeful activities that market forces may intervene with to advance the development, production, supply, and distribution of global goods for health, making essential medical products more affordable, accessible, innovative, sustainable and quality assured.

Over the past two decades, an increasing number of governments, international organizations, multilateral partnerships, and non-governmental organizations have resorted to market-shaping interventions, which Gavi is one of the international organizations to employ a lot. For example, Gavi revised its supply and procurement strategy in 2011 with the strategic goal of shaping the market for vaccines and other immunization commodities and applied its market shaping strategy on the development, production, supply, and distribution of 15 vaccines, such as COVID-19 vaccine, and two relevant immunization products based on their market-shaping strategy, which makes it possible for more people in developing countries to benefit from the relevant products within constrained health budgets [11, 12]. The Global Fund also has adopted market shaping strategies to halve the cost of antiretroviral drugs and create the Affordable Medicines Facility-malaria, a malaria medicines subsidy, to rapidly increase access to low-cost, high-quality artemisinin-based combination therapies and decrease the use of oral artemisinin mono-therapies [6]. All similar successful practices have proven the effectiveness of market shaping strategy in maximizing the impact of investments. However, as of now, the application of market-shaping strategies for essential medical products is mainly concentrated on limited types of products, such as vaccines and medical products for COVID-19 and malaria, which lacks the consideration of the whole product life cycle and combined use of multiple interventions, which is still far from satisfactory [1, 6, 12]. Given the urgent need to scale up the provision of public goods for health within constrained health budgets, market-shaping strategies should be more widely and creatively undertaken to accelerate access to essential products in developing countries. In this article, we draw on existing market-shaping strategies of international organizations, multilateral partnerships, and nongovernmental organizations to derive different sets of market-shaping activities and their associated outcomes.

HIV/AIDS has been a major public health challenge for decades, and there were approximately 38.4 million people living with HIV in 2021 globally, compared to about 26.0 million in 2000 [13]. HIV infections and AIDS-related deaths are largely determined by the availability of HIV products and services, and the development and supply of HIV/AIDS products need to be enhanced by market-shaping strategies in particular. Here, signs are worrying as the expansion of HIV testing and treatment services stalls [14]. With wide disparities in access to treatment and prevention among key and priority populations, helping developing countries strengthen national HIV testing programs has been a task requiring more creative efforts [15, 16]. The World Health Organization (WHO) recommends that countries adopt self-testing as part of a differentiated and comprehensive approach to HIV testing services. It is well known that HIV testing programs are at a critical juncture and new strategies need to be explored to leverage resources to efficiently and effectively reach the remaining undiagnosed people living with HIV [17, 18]. At the same time, relevant research has also shown that HIV self-testing has the potential to increase the uptake of HIV testing compared to standard HIV testing services, contributing to effective HIV treatment and prevention [19, 20].

To meet the medical needs of AIDS patients in developing countries in the post-COVID era, in July 2022, the Clinton Health Access Initiative (CHAI) and MedAccess, a social enterprise based in the UK, in partnership with Wondfo Biotech Co., Ltd. (Wondfo), a Chinese diagnostics company jointly announced a historic pricing agreement for Wondfo’s new HIV self-test, enabling a more effective and equitable global response to HIV prevention and treatment. This is the first-ever volume-guaranteed market-shaping intervention for an HIV self-test to approach price parity with conventional HIV tests ($0.80), giving countries more flexibility in expanding the use of HIV self-testing, which is also a practice and booster of the WHO Consolidated Guidelines on HIV Testing Services. Therefore, we use the HIV test kit as an example to identify the key determinants or enablers of market shaping strategies in boosting access to essential health commodities. The key learnings from this case demonstrate that a catalytic mechanism with active market creation and commitment is critical to bringing more affordable and quality-assured health commodities to high-burden countries.

## A conceptual framework

**Fig. 1:**
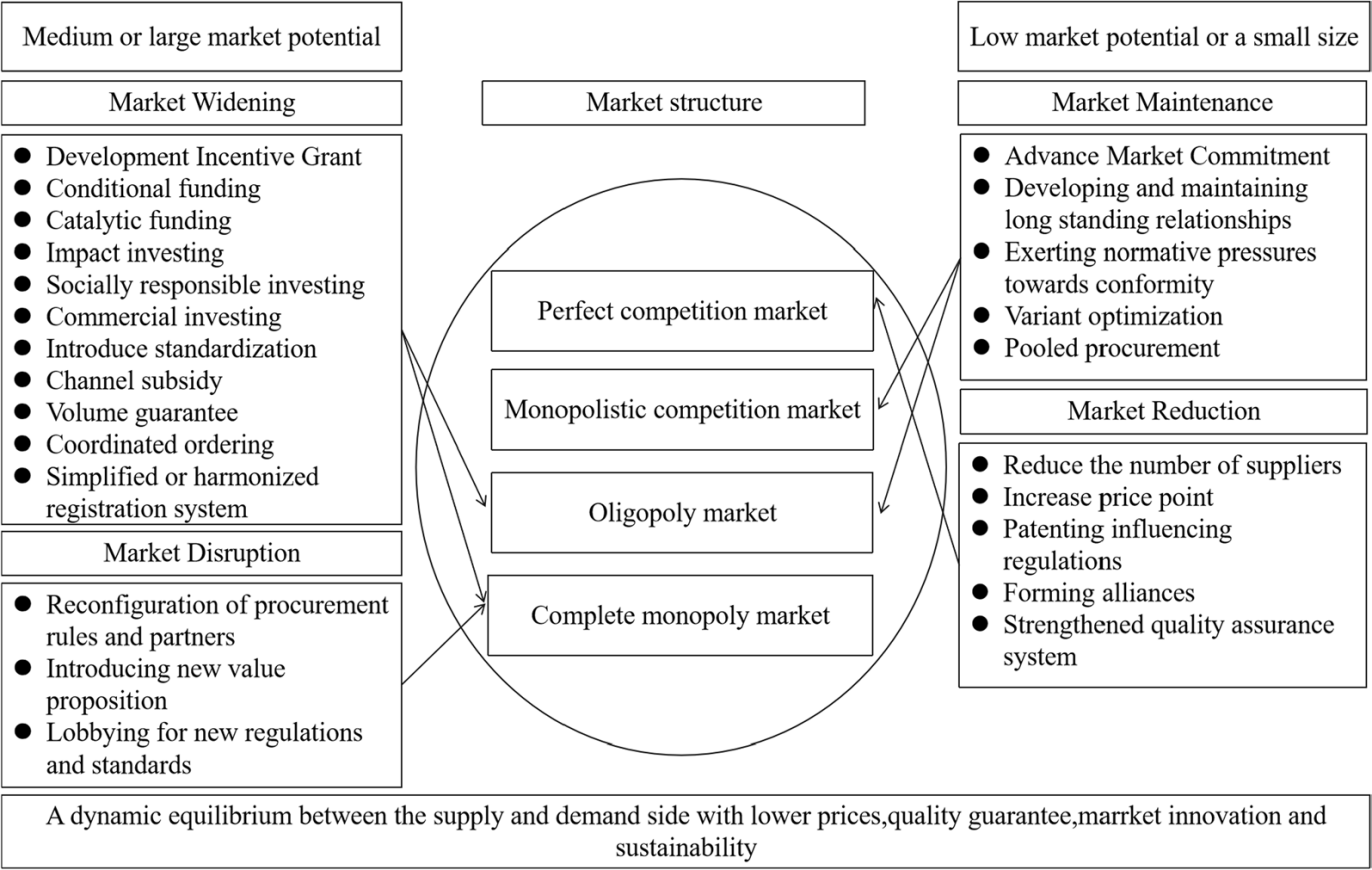
Market-shaping strategy framework

We propose a strategic market-shaping intervention framework to be referenced by key stakeholders, such as governments, international organizations, multilateral partnerships, and NGOs (see Fig. 1). As such, the key is to adopt market shaping techniques, which can be classified as market expansion, market disruption, market maintenance, and market contraction [9].

We also propose that those stakeholders should take it upon themselves to be the main drivers to shape or script the market. More attention should be given to the market structure to which policymakers are required to adapt market-shaping strategies given the classification of market potential. According to the structure-conduct-performance (SCP) model established by Bain and Scherer [21, 22], market structure determines firm conduct, which would determine performance. Clearly, market structure is an indispensable element to be reckoned with. Whatever the market structures, it is no easy job to strike a  proper balance between affordability, market innovation, and sustainability in the oligopolistic or monopolistic market. Therefore, market widening/ expansion or disruption strategies are viable options to shape the market. For the perfect competition with low market potential or of a small size, market reduction/contraction strategies are recommended to address market failures to sustain or strengthen innovation and sustainability. While for the monopolistic competition , market maintenance strategies may be a better choice to script the market.

For essential medical products, market failures may exist across the product life cycle, ranging from research and development, standardization and regulation, manufacturing, commercialization, and procurement to supply chain management. Therefore, a set of market shaping tools are needed to keep a dynamic balance between supply and demand (see Table 1). In many cases, comprehensive or combined use of multiple and multilevel interventions is strongly recommended. For example, market shaping for antimicrobial resistance drugs, and diagnostic reagents could be applied by global, regional, national, and subnational organizations at the same time and in various manners. In order to better respond to future pandemics, what is desired is the creation of an end-to-end ecosystem that delivers equitable research, development, manufacturing, and access to epidemic countermeasures, grounded in a common goods approach that responds to local needs, with equity built in from research to access [5].

**Table 1:** Main market shaping tools to support interventions

	Research and development	Normative and regulatory	Manufacture and commercialization	Procurement and supply management	Introduction and scale
*Donor funding*					
Prize	✓				
Development incentive grant	✓	✓	✓		
Advance market commitment	✓	✓	✓	✓	
Product subsidy				✓	✓
*Guarantees*					
Volume guarantee		✓	✓	✓	✓
Procurement guarantee				✓	✓
Payment guarantee				✓	✓
*Debt/Equity*					
Working capital facility			✓	✓	✓
Impact investment	✓	✓	✓	✓	
*Other*					
Regulatory incentive	✓	✓			
Reconfiguration of procurement rules and partners	✓		✓	✓	
Developing and maintaining long standing relationships			✓	✓	✓

## Market shaping for HIV self-testing

Since there are often high transaction costs associated with a multiparty deal, preparations need to be made as follows: a strong case for the product or service with a direct impact on patients and/or value for money; a solid demand forecast for the product or service based on price sensitivity; a compelling explanation of how the financial instrument(s) and other interventions will address important supply and demand constraints relative to the alternative without compromising the overall, longer-term health of the market; and identification of potential financing partners with reference to the financial instruments, expected deal size, and risk profile. In the case of the HIV self-test supplied by Wondfo Biotech Co., Ltd, CHAI hoped to reduce the price to $1 per test or less in prospective companies through market-shaping strategies to maximize access in developing countries without jeopardizing the overall, longer-term health of the market.

The HIVST mainly consists of a spring-loaded retractable safety lancet, packaging materials, volume, and packaging of buffers and other accessories, while some professional-use RDTs use standard lancets. CHAI found that the use of a safety lancet alone could add between $0.011 and $0.04 per test only. In terms of packaging materials, professional-use tests are typically packaged in boxes of 10–250 units, whereas HIVST products are individually packaged, including instructions for use and waterproof packaging. This suggests that individually packaged tests appear to be the largest cost driver. However, this could be addressed by bulk packaging. In terms of volume and packaging of buffers and other accessories, HIVST products require individual packaging of buffers and may require an additional volume. For tests requiring multiple buffers or large quantities of buffers, this would be a cost driver. Packaging appears to be the largest cost driver identified through the teardown exercise.

In recent years, advocacy and evidence generation have led the WHO and PEPFAR to update their HIVST guidelines to include stronger recommendations for direct facility-based distribution. The PEPFAR 2020 & 2021 Country Operational Plan [23], called for a reduced price for HIVST of $1 per test or less, with a commitment to support the targeted use of HIVST in facilities. There was preliminary evidence that at this price, HIVST, if appropriately integrated into workflows, would increase testing among priority populations such as men and adolescents and relieve health workers of the need to perform each test individually. During the COVID-19 outbreak, HIVST also enabled countries to maintain HIV testing services while limiting the number of clients in facilities, which more effectively catalyzed HIVST uptake [24]. While overall HIV testing was significantly disrupted by the COVID-19 pandemic, HIVST distribution volumes have increased significantly in many countries over the past few years. WHO prequalified HIV self-tests have already been made available. However, unlike single HIV tests for professional use in health facilities, many governments are unable to procure them they would like due to the high price [25, 26].

Therefore, CHAI and MedAccess worked together to develop strategies to stimulate the supply of high-performance, quality-assured but affordable products in CHAI-supported countries by increasing demand visibility and improving the exchanges between manufacturers and purchasers. Meanwhile, they sought to minimize the cost of HIV self-testing by implementing warranty approaches, de-risking manufacturers’ production planning, and improving price transparency. In July 2022, with the support of CHAI and MedAccess, Wondfo agreed to make this newly WHO prequalified and blood-based HIV self-test available to public sector purchasers in 140 low- and middle-income countries for $1 ex-factory. This is 50% less than the current leading HIV self-test (OraQuick at $2) and more than 30% less than the lowest-priced test to date (Abbott at $1.50).

A confluence of multiple factors led to the collaboration between CHAI, Wondfo, and MedAccess. Given the widespread uptake of self-testing during the COVID-19 pandemic, CHAI quickly identified a common interest in bringing a lower-cost HIVST to the public sector market and then facilitated an introduction to MedAccess. The main process in which three parties played their due roles is as follows: MedAccess sent out a formal request to relevant diagnostic suppliers, including Wondfo, to submit proposals to introduce a $1 HIVST; Wondfo then provided product dossiers, performance analysis, and company policies while CHAI provided market intelligence and other supportive documents. Since Wondfo submitted the most compelling application, negotiations on a possible volume guarantee between Wondfo and MedAccess kicked off. Finally, CHAI, Wondfo, and MedAccess reached a 5-year volume guarantee agreement under which $1 price applies to all public sector purchasers in 140 low-and-middle-income countries, a record low price compared to the $2 HIVST made by OraQuick and the $1.5 one by Abbott. CHAI then awarded a 5-year grant to support the launch of $1 HIVST in 6–10 countries, with a 12-month engagement in each country to expedite approval for use (including registration, validation, field evaluations/pilots), ensure supplier compliance and business performance monitoring, and leverage complementary work. CHAI also helps Wondfo reach out to procurement groups, while Wondfo is responsible for monitoring product sales under the volume guarantee and provides quarterly reports to partners on shipments, orders, and inquiries. If sales are less than the agreed upon annual target, CHAI will purchase the shortfall from Wondfo at the end of the year as per the terms of the contract.

## Expected outcomes of market shaping in HIV self-testing

According to the World Health Organization (2011) [27], access to essential medical products is determined by five variables: appropriateness, availability and affordability, quality assurance, health security, and continuous innovation. Due to the market failure of essential medical products, it is imperative to balance these variables to promote access and maximize global welfare through market shaping strategies. In the case of HIV self-testing, evidence, and modeling have shown that the use of HIVSTs as facility-based screening tools would increase testing among priority populations while dramatically reducing the amount of time health workers spend on HIV testing [28–30]. There have been  six WHO prequalified HIVST kits so far [31]. The volume guarantee implemented by CHAI contributes significantly to the enhancement of affordability and availability of WHO prequalified HIV self-tests. It allows more people to benefit from limited charitable funds to use HIV self-testing products, which are available at only $1 due to this market shaping practice. This may also stimulate other companies to pursue lower prices, quality guarantees, and market innovation in HIV self-testing products as the market is widening.

In collaboration with an organization in Malawi, CHAI sought to examine the use of facility-based health services by under-reached populations and found that many people in need of HIV testing attend health facilities but are not being offered HIV testing [28, 29]. HIVST offered in facilities is acceptable, increases testing uptake, results in similar positivity rates to standard HTS, and increases new identifications [29]. HIVST led to a threefold increase in the proportion of outpatients tested for HIV compared with standard provider-initiated testing [29]. For a lower price, countries could fully scale up HIVST for use in current priority distribution channels and initiate widespread use as a screening test in facilities. In the framework of COVID-19, people living with HIV in developing countries have health and financial problems. Equality of human welfare will benefit from this policy.

Moreover, lower price for HIVST makes digital interventions more cost-effective, which would contribute to the treatment and prevention of HIV. Ongoing programmatic interventions are very important to the effectiveness of the program. Digital interventions for HIVST have been proposed by WHO to address the following challenges of the HIVST because the individual self-test cannot be appropriately tracked and not all positive cases are linked to care [32, 33].

## What can we learn from the framework and market shaping for the HIV self-test?

In the marketplace of essential medical products, a single market failure at some point may prevent life-saving products from reaching people in need, and addressing these failures and promoting better health outcomes for the vulnerable population thus become the goals of market-shaping strategies. Market shaping may modify or optimize existing market structures by minimizing transaction costs, increasing market knowledge, and balancing supplier and buyer risks, resulting in efficiencies that improve health outcomes [6]. However, there are some issues to be reckoned with in market shaping interventions, such as how to strike a proper balance between innovation and affordability of badly needed health products, how to change the price elasticity of demand through substitution of products, etc. In a closely interconnected ecosystem, developers, manufacturers, purchasers, and distributors must work together to shape the market across the product life cycle.

Market-shaping has played an increasingly important role in promoting access to HIV self-testing in developing countries. Similar to these products, various regional governments and other international organizations can also use other market shaping interventions to promote access to essential medical products. More importantly, global, regional, national, and subnational efforts in different stakeholders could be coordinated and integrated into the practices of market-shaping to solve the market failures of a specific essential medical product in parallel, so as to maximize the impact of market-shaping in limited resources.

We should tailor our approaches to different markets. In the event of a public health emergency, all possible market expansion and disruption strategies need to be adopted by multilevel and multisectoral stakeholders to contribute to the research and development of relevant essential medical products. Here are some considerations for pushing forward the process:

First, there are different market-shaping strategies depending on the objectives and specific problems to be addressed. Market-shaping strategies normally focus on market outcomes that include a more thorough knowledge of the long-term influence of an international organization's strategic activities on a market, in contrast to earlier market-strategy conceptualizations that often emphasize the firm’s role in such strategies. This gives a broader picture of the effects that conventional market techniques always overlook. The post-COVID era will be filled with multiple challenges and uncertainties in the development and supply of essential health products, and more innovative mechanisms will be entailed. In addition to public donations, a diversified contribution, particularly from the private sector, will be required to address future pandemics and health challenges, leading to the development of market-based financing mechanisms to support the sustainable provision of essential public health commodities in developing countries.

Second, markets exhibit varying levels of stability over time. The practice that stability and instability can be managed by market-shaping actors has gained traction in recent decades [34]. However, when market-shaping techniques are designed, it is a must to take into account the short-term vulnerability of the market, which entails an incubation period and the constant evaluation and monitoring of the performance before the scale-up.

Third, market-shaping strategies rarely occur in isolation. Market conditions are bound to change as a result of the interactions between stakeholders. Due to the different goals and visions of market participants, market interventions normally occur simultaneously and in an interwoven manner. An organization pursuing market maintenance or market reduction tactics will encounter another market actor attempting to disrupt the market. In this scenario, balancing multiple market-shaping strategies becomes a precondition for success [35].

Fourth, a set of analytical tools and market shaping strategies can help diagnose and intervene in the root causes of a shortcoming by examining market actors, their interactions, or their regulatory systems. Multiple failures and shortcomings may stem from the same root causes. Access to essential health products with a global public health nature is  certain to face more market failures or shortcomings, requiring both push and pull mechanisms to create an enabling environment. Pricing, patents, and volume of procurement will be the entry points for future interventions in the healthcare market. And the process of regulatory harmonization in some regions will also facilitate access to essential health commodities and the configuration of an enlarged market of public goods for health.

## Conclusions

Market shaping alone does not address the multitude of challenges of accessing essential medical products  in developing markets. Ongoing programmatic interventions, such as training of health care providers, procurement of health products, and strengthening of the supply chain by the global health community to implement and effect change are very important and necessary to ensure the effectiveness of market shaping strategies in essential medical products. In designing a market-shaping strategy, policymakers need to bear in mind that health markets are dynamic, interactive, and constantly evolving, and particular strategies and activities can have an impact on all market components, including market actors and elements. This makes it more important to choose the right and appropriate strategies and to adapt them over time according to the existing resources and funds. In the future, more comprehensive market shaping strategies, including the strategic use of market expansion, market disruption, market maintenance, and market contraction alone or together, deserve to be explored by different stakeholders to make the intervention more efficient and effective. More innovation and practice in market-shaping are desired to narrow the gap and better meet the fast-growing demand in developing countries, which could be a game-changer in reshaping the life cycle of global public goods for health and further accelerating accessibility in the long term.

## Data Availability

Not applicable.

## Abbreviations

WHO: World Health Organization
UNICEF: United Nations Children's Fund
UNFPA: United Nations Population Fund
Global Fund: The Global Fund to Fight AIDS, Tuberculosis and Malaria
Gavi: Gavi, the Vaccine Alliance (previously the Global Alliance for Vaccines and Immunization)
CHAI: The Clinton Health Access Initiative
Wondfo: Wondfo Biotech Co., Ltd.
HIVST: HIV self-testing
